# Cohabitation and roommate bias of symbiotic bacteria in insect hosts

**DOI:** 10.1111/mec.15295

**Published:** 2019-11-22

**Authors:** Sharon E. Zytynska

**Affiliations:** ^1^ Department of Evolution, Ecology and Behaviour Institute of Integrative Biology University of Liverpool Liverpool UK

**Keywords:** adaptation, community ecology, insects, species interactions, symbionts

## Abstract

Symbiotic interactions between insects and bacteria have long fascinated ecologists. Aphids have emerged as the model system on which to study the effect of endosymbiotic bacteria on their hosts. Aphid‐symbiont interactions are ecologically interesting as aphids host multiple secondary symbionts that can provide broad benefits, such as protection against heat stress or specialist natural enemies (parasitic wasps and entomopathogenic fungi). There are nine common aphid secondary symbionts and individual aphids host on average 1–2 symbionts. A cost‐benefit trade‐off for hosting symbionts is thought to explain why not all aphids host every possible symbiont in a population. Both positive and negative associations between various symbionts occur, and this could happen due to increased costs when cohosting certain combinations or as a consequence of competitive interactions between the symbionts within a host. In this issue of *Molecular Ecology*, Mathé‐Hubert, Kaech, Hertaeg, Jaenike, and Vorburger (2019) use data on the symbiont status of field‐collected aphids to inform a model on the evolution of symbiont co‐occurrence. They vary the effective female population size as well as the rate of horizontal and maternal transmission to infer the relative impact of symbiont‐symbiont interactions versus random drift. Additional data analysis revisits an association between two symbionts in a fruit fly species using a long‐term data set to highlight that such interactions are not limited to aphids.

Aphids are small, soft‐bodied insects that feed on plant‐phloem sap and are major crop pests as they can transmit plant viruses and reduce crop yield. They reproduce asexually during the warm summer months, with each female producing hundreds of clonal offspring (Figure 1). Telescopic generations, where an offspring is born already pregnant, enhances the population growth potential of these insects leading to exponential growth rates under favourable conditions. Interactions with symbiotic bacteria can also benefit aphid survival and population growth. However, only 40%–60% of aphids in a population are infected by even the most prevalent secondary symbiont (Zytynska & Weisser, 2016) indicating that there must be some way in which host aphids lose and gain symbionts. Aphid symbionts are predominantly vertically transmitted from mother to offspring during reproduction, with transmission failure rates ranging from 0% up to 40% in the field depending on the symbiont species, other hosted symbionts, and host genotype (Rock et al., 2018). Horizontal transfer of symbionts among aphids can also occur during sexual reproduction, by parasitic wasps when ovipositing eggs into the aphids, or even through infected honeydew (reviewed in Zytynska & Weisser, 2016). To maintain coexistence of symbiont‐infected and uninfected aphids, a recent mathematical model showed that horizontal transmission rates via parasitic wasps must be low, otherwise all aphids become infected (Zytynska & Venturino, 2019); this model assumed 100% maternal transmission rates and no migration. A new meta‐analysis supports the theory that symbionts are costly to host in aphids and other sap‐feeding insects (Zytynska, Thighiouart, & Frago, 2019). However, the effect sizes associated with these interactions varied widely depending on the species and genotype of both symbiont and aphid. Much of this work has been achieved through controlled laboratory experiments, which compared clones of aphids that harboured a symbiont compared to those that were cured of these symbionts. Continued experiments to further understand the impact of cohosting multiple symbionts is encouraged.

**Figure 1:**
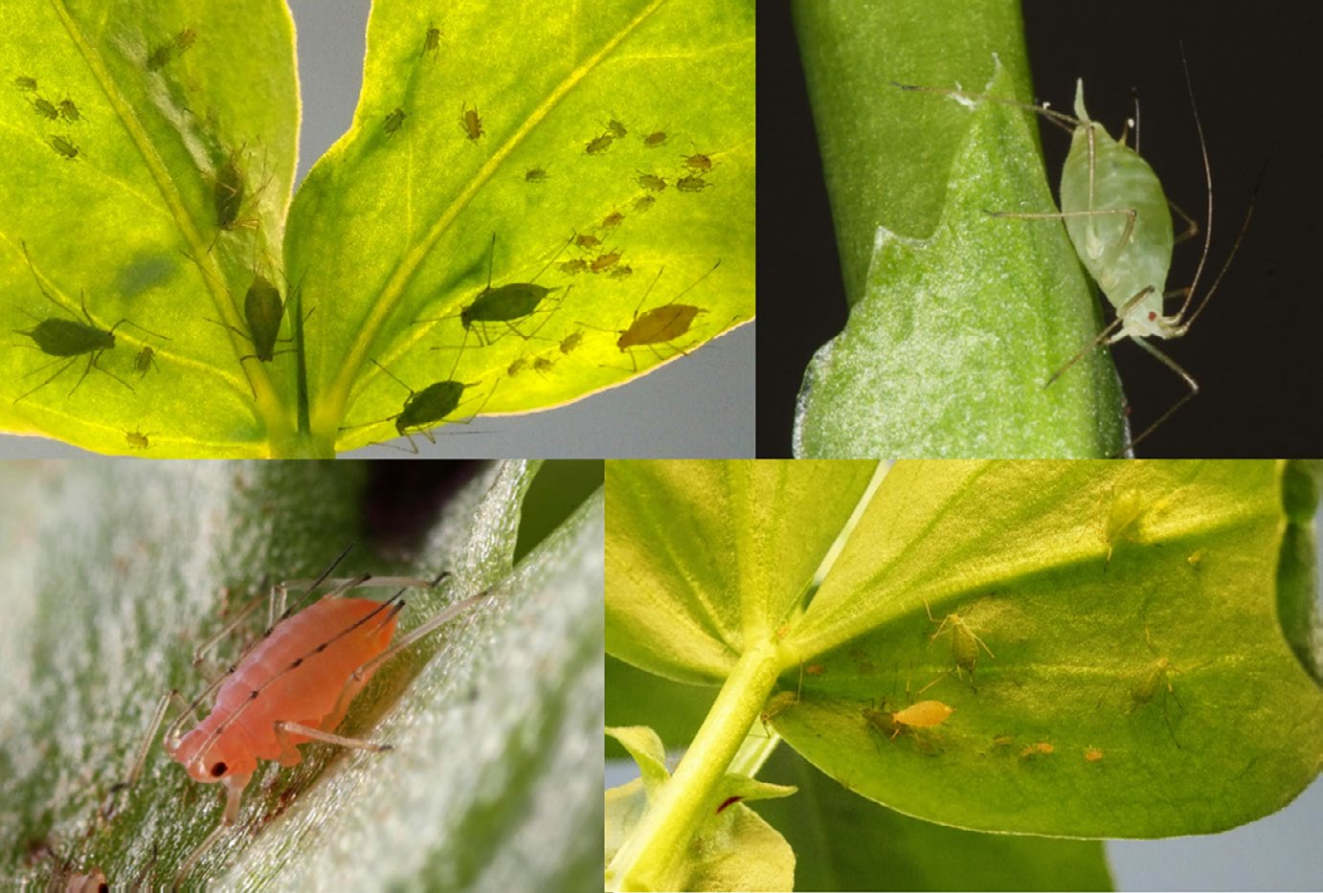
Pea aphids (*Acyrthosiphon pisum*) are found in two common colour morphs (pink and green), and also form host‐associated races that feed on different host plant species. A universal host‐plant of these aphids is the broad bean plant (*Vicia faba*), on which the pictured aphids are feeding. Photo credit: Hugo Mathé‐Hubert et al

Recent interest in aphid symbionts has developed beyond the initial “what do they do?” question towards more community ecology questions based on their role in natural systems. Currently, this is rather theoretical with a number of reviews describing potential effects within multispecies networks (McLean, Parker, Hrček, Henry, & Godfray, 2016), the role of plant and natural enemy diversity (Zytynska & Meyer, 2019), and the challenges for biocontrol (Vorburger, 2018). The building evidence that host‐symbiont dynamics are strongly influenced by maternal and horizontal transmission rates, host and symbiont genetic variation, and the wider interacting community, highlights the need to focus efforts on combining laboratory experimental work with field studies. One step forward is provided by Mathé‐Hubert et al. (2019) in this issue of *Molecular Ecology*. These authors explore the role of drift and symbiont‐symbiont interactions to explain associations between various symbiont species in field populations. This is imperative since previous work often assumed associations to be driven by symbiont‐symbiont competition/ facilitation that prevents/promotes co‐occurrences without considering other mechanisms.

Mathé‐Hubert et al. (2019) analysed a European data set on symbiont occurrence from 498 pea aphids (*Acyrthosiphon pisum*), identifying seven symbiont species: *Hamiltonella defensa*, *Regiella insecticola*, *Serratia symbiotica*, *Rickettsia*, *Rickettsiella*, *Spiroplasma* and X‐type (now *Fukatsuia symbiotica*). They found similar average numbers of symbionts within aphids collected from two different host plant species (0.97 symbionts per aphid on *Trifolium* spp. and 0.77 on *Medicago sativa*, with similar sample sizes). However, associations between different symbionts, when an aphid hosted multiple, varied across host plant species with more associations present within aphids collected from clover (*Trifolium* spp.) than alfalfa (*M. sativa*). Consistent associations across the data set included a negative association between *H. defensa* and *R. insecticola* and a positive one between *H. defensa* and X‐type (previously identified, reviewed in Mathé‐Hubert et al., 2019; Zytynska & Weisser, 2016). The majority of negative associations occurred among the more abundant symbionts, leading to a negative correlation between the prevalence of a particular symbiont and the number of coinfecting symbionts to be greater than expected under random assortment. This overall trend was also significant for *H. defensa*, for *R. insecticola* and for *S. symbiotica*. However, many of the less abundant symbiont species experienced a positive association with the more abundant species, suggesting that they might need the other symbionts to persist and selection maintains such associations. The impact of drift on these interactions would be to introduce random variation into the frequency of associations, and as such could also have created these associations to start with. The authors conclude that while drift may have played some part, the consistency of these associations across continents (Europe and N. America) means that most (if not all) are probably driven by symbiont‐symbiont interactions.

The second part of the study by Mathé‐Hubert et al. (2019) focused on the within‐species variation of the *Spiroplasma* symbiont. They identified three main genetic clades (clusters) to show that while there was no variation in strain frequency across host plant species, the frequency of these clades varied strongly due to the presence of other symbionts. For example, *Spiroplasma* from clade 2 was more frequent in aphids that cohosted other symbionts, and clade 3 *Spiroplasma* was less frequent if the aphid cohosted *H. defensa*. This highlights the necessity to consider the co‐occurrence of aphid and symbiont genotypes as well as variation across host species, and can also inform on rates of horizontal transmission as suggested by Mathé‐Hubert et al. (2019). While this is not a new idea (Ferrari & Vavre, 2011), we now have the knowledge and ability to design field experiments to transfer theoretical and controlled experimental work to realistic systems. Mathé‐Hubert et al. (2019) also revisited an interaction between *Wolbachia* and *Spiroplasma* in *Drosophila neotestacea* (Figure 2) as the third and final analysis in the paper. Based on a long‐term data set detailing this association, Mathé‐Hubert et al. (2019) applied their model to the data and found that a decline in frequency of this association was not due to drift. In fact, when accounting for drift a strong symbiont‐symbiont interaction was still detected. A previous study by Fromont, Adair, and Douglas (2019) showed that while *Wolbachia* has a positive effect on *Spiroplasma* density, this does not happen vice versa—yet *Wolbachia* does benefit from the pathogen‐fighting function of *Spiroplasma*. Overall, these set of analyses reveal that symbiont‐symbiont interactions are abundant and not limited to one particular insect group. With increasing efforts in detecting symbiosis across multiple insect groups, future work could uncover such effects in a wider set of species.

**Figure 2:**
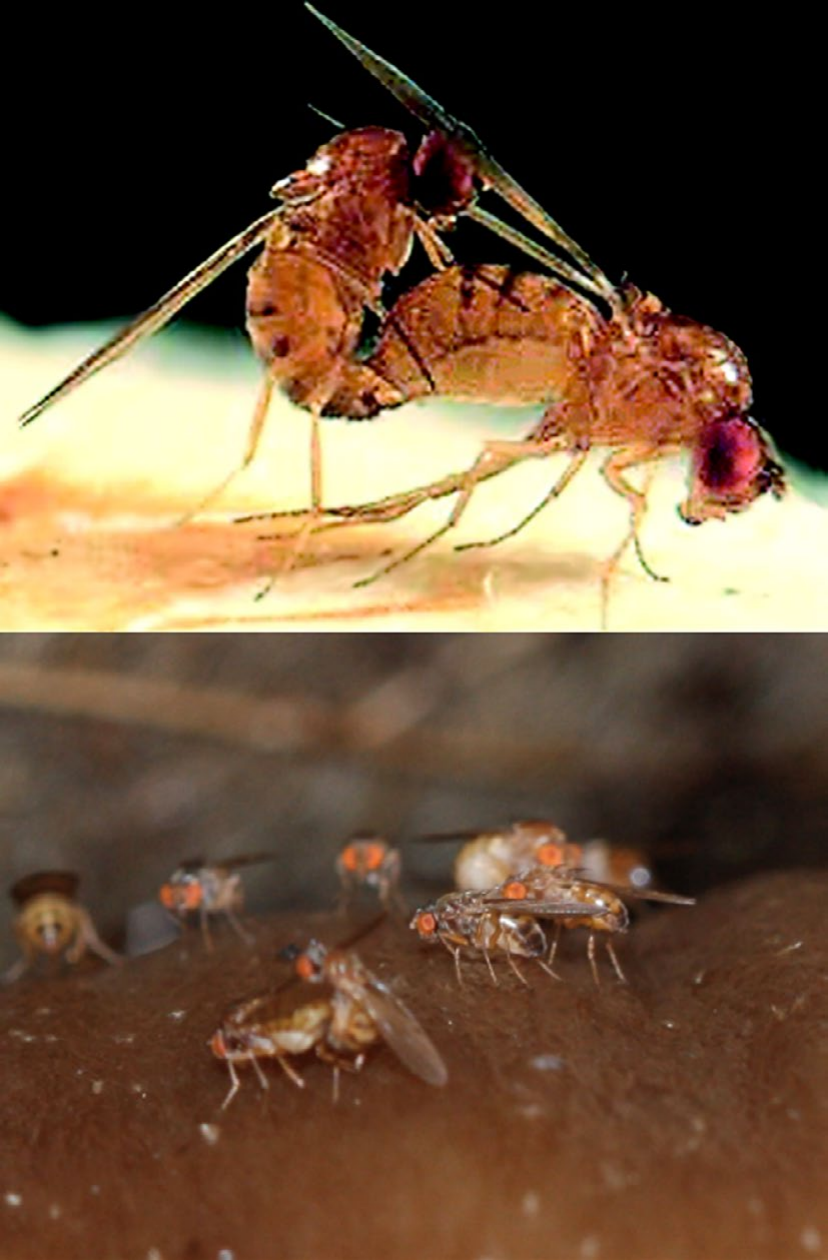
The fruit fly *Drosophila neotestacea* breeds on mushrooms and is often studied for their interactions with *Howardula* nematode parasites. Here, they are studied for their interactions with endosymbiotic bacteria *Spiroplasma* and *Wolbachia*. Photo credit: Hugo Mathé‐Hubert et al

Research on insect‐symbiont interactions is currently taking an exciting leap forward. We are beginning to use experimental and field survey data to inform models on which to create future experimental hypotheses as here by Mathé‐Hubert et al. (2019). One important step is to incorporate the cohosting of multiple symbionts into our models and experiments. It follows that there is great potential for uncovering novel microbe‐mediated functions in ecological systems, with the term “holobiont” often used to describe an individual and all its interacting species (as an ecological unit). The societal impact of this knowledge is also high, with natural enemies often used as a main biocontrol method in glasshouses – and where the spread of protective symbionts in pest populations can have major consequences for biocontrol efficiency (Vorburger, 2018).
